# Electronic control of DNA-based nanoswitches and nanodevices†
†Electronic supplementary information (ESI) available: Experimental procedures. See DOI: 10.1039/c5sc03694a



**DOI:** 10.1039/c5sc03694a

**Published:** 2015-11-12

**Authors:** Simona Ranallo, Alessia Amodio, Andrea Idili, Alessandro Porchetta, Francesco Ricci

**Affiliations:** a Chemistry Department , University of Rome Tor Vergata , Via della Ricerca Scientifica , Rome 00133 , Italy . Email: francesco.ricci@uniroma2.it; b PhD School of Nanotechnology , Department of Physics , University of Trieste , Trieste , Italy

## Abstract

Here we demonstrate that we can rationally and finely control the functionality of different DNA-based nanodevices and nanoswitches using electronic inputs. To demonstrate the versatility of our approach we have used here three different model DNA-based nanoswitches triggered by heavy metals and specific DNA sequences and a copper-responsive DNAzyme. To achieve electronic-induced control of these DNA-based nanodevices we have applied different voltage potentials at the surface of an electrode chip. The applied potential promotes an electron-transfer reaction that releases from the electrode surface a molecular input that ultimately triggers the DNA-based nanodevice. The use of electronic inputs as a way to finely activate DNA-based nanodevices appears particularly promising to expand the available toolbox in the field of DNA nanotechnology and to achieve a better hierarchical control of these platforms.

## Abstract 2

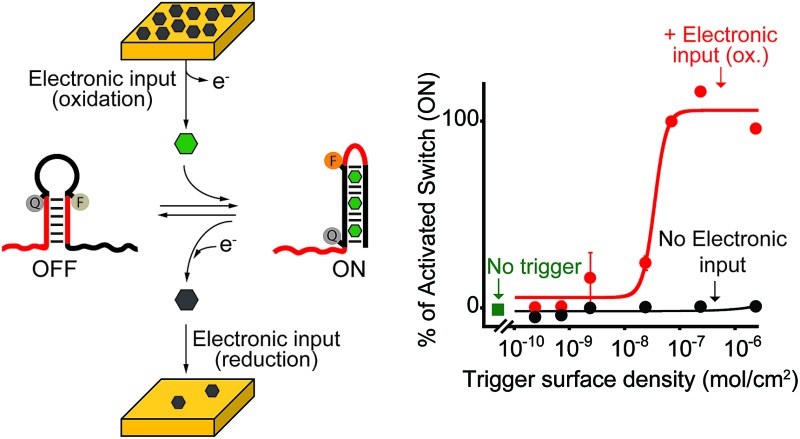
Here we demonstrate that we can rationally and finely control the functionality of different DNA-based nanodevices and nanoswitches using electronic inputs.

## Introduction

The growing and exciting field of DNA nanotechnology, where synthetic nucleic acids are rationally engineered and designed to build novel responsive nanomachines or functional nanodevices, represents one of the most interesting examples of bio-inspired technologies.^1^ The majority of these DNA-based nanodevices rely on a common basic mechanism: a target molecular input is recognized by a DNA probe sequence and the binding event is coupled to a mechanical motion or an output signal.^2^ Even the most complex functional DNA-based nanostructures (*i.e.* origami) are thus based on the use of relatively short DNA sequences (or nanoswitches) that in the presence of a specific molecular input undergo binding-induced conformational changes or DNA-based reaction and, by doing so, confer to the nanostructure an useful function.^3,4^ Despite the advancements achieved in this field, the need to have a better control of such nanodevices remains still partially unmet. To fully exploit these platforms it would thus be crucial to find new strategies to trigger and activate their function in a highly controllable fashion.

Since the revolutionary discoveries of Volta, Faraday and other pioneer electrochemists,^5^ the possibility to control redox reactions through an electronic input (applied voltage) has represented one of the major breakthrough in the history of chemistry. It is now more than 200 years that electrochemistry has been applied for a wide range of applications from energy production^6^ to industrial manufacturing^7^ and sensing.^8^ Because of the low cost of instrumentation, possible miniaturization and high level of control, electrochemistry might also represent an interesting opportunity for novel bio-technological applications. Redox reactions are in fact routinely used in Nature to activate, regulate and control a wide range of biological pathways and reactions (such as photosynthesis and energy storage/release).^9^ In a similar way, electrochemistry could thus be applied to modulate bio-inspired tools and devices.^10–13^ Despite this, the possibility to use electronic inputs to control DNA-based nanodevices has seen very little application to date.^14^

**Fig. 1:**
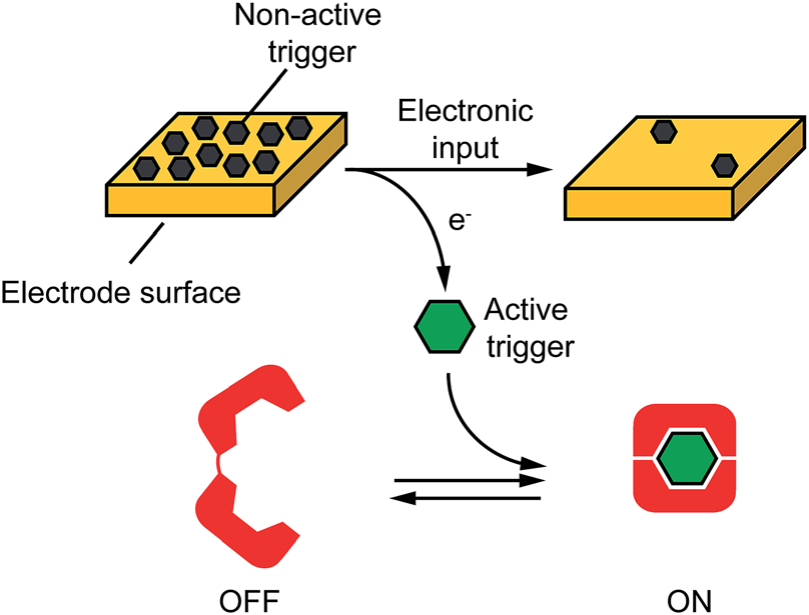
DNA nanodevices or nanomachines are usually based on responsive DNA sequences (red) that, upon binding to a specific molecular input (green trigger) undergo a conformational-switch or a specific reaction that is converted into a signal. Here we demonstrate that we can electronically activate similar responsive DNA-based nanodevices. In our approach a non-active trigger (grey) is coated on the surface of a chip electrode. The electronic input promotes an electron transfer exchange reaction that activates the trigger and releases it from the chip's surface. The active trigger is then able to bind the DNA nanodevice activating it. We used here four different DNA-based nanodevices: two conformational-change DNA nanoswitches responding to metal ions (Hg(ii) and Ag(i)), a stem-loop DNA molecular beacon responding to a specific DNA sequence and a Cu(ii)-responsive DNAzyme.

Motivated by the above arguments, here we propose an approach to electronically control a wide range of DNA-based nanodevices. We did so by controlling, through an electronic input, electron transfer across an electrode-solution interface thus promoting redox reactions in a highly controllable fashion. More specifically, as the electronic input we used here a voltage potential applied at the surface of an electrode chip. Such applied potential promotes an electron-transfer reaction at the electrode-solution interphase leading to the release of a molecular input that ultimately triggers a DNA-based nanodevice in solution (Fig. 1). To demonstrate the versatility of our approach we have used here four different model DNA-based nanodevices or nanoswitches that can be activated by different molecular inputs.

## Results and discussion

**Fig. 2:**
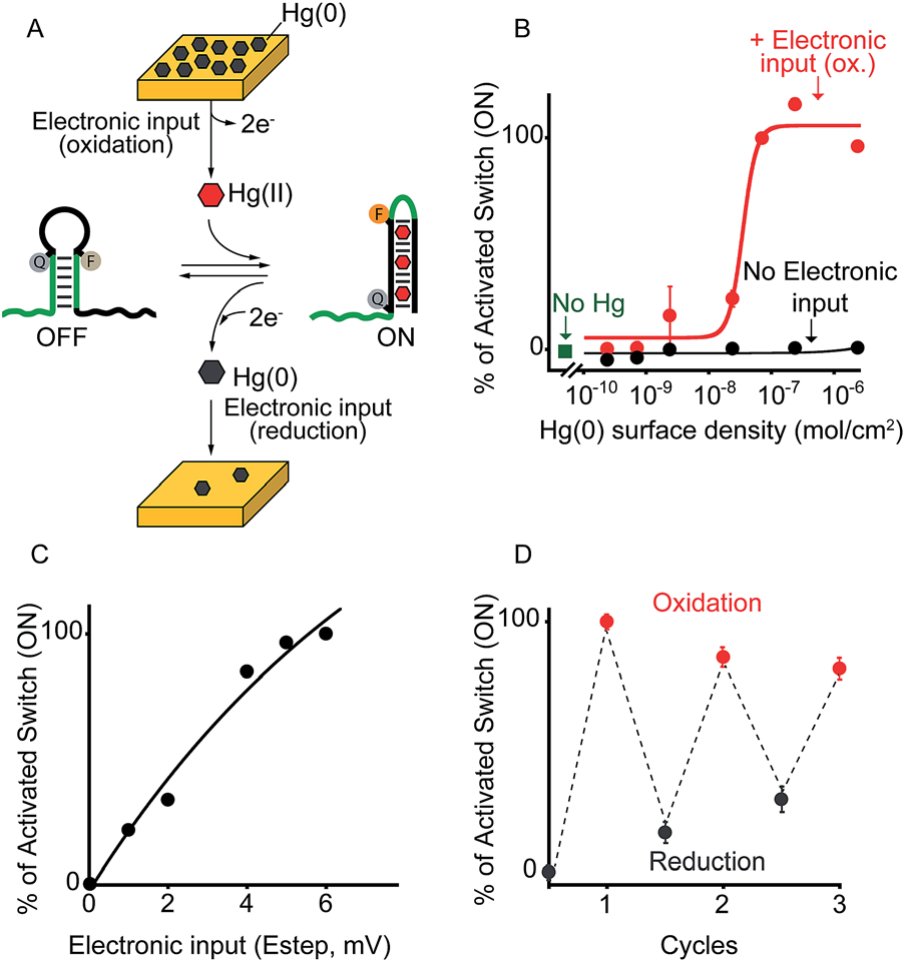
Electronic control of a Hg(ii)-responsive DNA-based nanoswitch. (A) Voltage-induced release of Hg(ii) ions from a gold chip coated with Hg(0) or deposition of Hg(0) to a gold chip from a Hg(ii) solution allows to control the nanoswitch's activation and inhibition respectively. (B) We can modulate the percentage of nanoswitch electronic activation by varying the density of Hg(0) on the chip (from 2.4 ± 0.2 × 10^–6^ to 2.4 ± 0.3 × 10^–10^ mol cm^–2^). Control experiments were performed using (i) Hg(0)-coated chips without applying the oxidation potential (no electronic input, black curve) and (ii) applying the oxidation potential to chips without Hg(0) coating (green square). (C) We can also modulate the Hg(ii)-responsive nanoswitch by using chips with fixed Hg(0) density (2.4 ± 0.2 × 10^–6^ mol cm^–2^) and by varying the step potential (*E*_step_) used during the oxidation scan. (D) By cyclically oxidizing Hg(0) (release from chip) and reducing Hg(ii) ions (depositing on chip) we can activate and inhibit our Hg(ii)-responsive nanoswitch in a reversible way. Here fluorescence measurements were performed in 50 mM sodium phosphate, 150 mM NaCl, pH 7.0 at 25 °C containing the Hg(ii)-responsive nanoswitch (10 nM). See ESI for experimental details.†

As a first proof-of-principle of our strategy, we demonstrate here that we can electronically trigger the conformational change of a DNA-based nanoswitch. To do this we have used a previously reported DNA-nanoswitch whose binding-induced conformational change can be triggered by Hg(ii) ions.^15^ The nanoswitch is designed to be in a thermodynamic equilibrium between two low-energy states: a non-binding (OFF) conformation that lacks the Hg(ii) binding sites and a binding-competent conformation (ON) that contains multiple T–T Hg(ii)-binding mismatches^15^ (Fig. 2A). The sequence is designed so that the non-binding state is more stable and only in the presence of Hg(ii) ions this equilibrium is pushed toward the binding-competent conformation, coupling recognition with a large conformational change. Because the nanoswitch is labelled with a fluorophore and quencher we can easily follow the conformational change of this nanoswitch by fluorescence measurements (Fig. S1†).

We can trigger the conformational change of such DNA-nanoswitch by using an electronic input. To do so we used a gold chip^16^ coated with a film of Hg(0) (Fig. 2A). By applying on this chip's surface an oxidative potential scan (from 0.2 to 0.65 V *vs.* Ag/AgCl) it is possible to promote an electron-transfer reaction that will lead to the oxidation of the deposited Hg(0) to Hg(ii) ions which, in turn, will diffuse to the bulk of the solution and trigger the conformational change of the DNA nanoswitch (Fig. 2A). Of note, by varying the density of Hg(0) on the chip surface from 2.4 ± 0.2 × 10^–6^ mol cm^–2^ to 2.4 ± 0.3 × 10^–10^ mol cm^–2^ (Fig. S2†) we can finely regulate the activation of the nanoswitch (Fig. 2B). A control experiment performed under the same experimental conditions but without applying the electronic input leaves the nanoswitch completely inactivated (Fig. 2B, black curve, no electronic input). Similarly, by applying an oxidative potential to a chip without Hg(0) coating results in no activation of the nanoswitch (Fig. 2B, green square). We can also modulate the electronic activation of the nanoswitch by varying the electronic input applied on the chip's surface. More specifically by using a chip with a fixed Hg(0) density (2.4 ± 0.2 × 10^–6^ mol cm^–2^) we have varied the width of the potential step during the oxidative scan (from 1 mV to 6 mV) thus producing a modulation of the activation of the nanoswitch (from 22 ± 1% to 100 ± 2% respectively) (Fig. 2C). The electronic strategy we propose to control DNA nanodevices is reversible and can achieve regeneration. We demonstrate this by cyclically releasing Hg(ii) ions and depositing Hg(0) through the application of oxidative (0.2 V) and reductive potentials (–0.3 V), respectively. By doing so we show that we can cyclically activate and inhibit the DNA nanodevice in a reversible way (Fig. 2D).

By using a similar approach we can also electronically activate a DNA-based switch whose conformational change can be triggered by the formation of multiple C–C Ag(i)-binding mismatches^15^ (Fig. S3†). To do so we employed chips produced by using silver-based conductive inks. By applying a potential scan to this silver-based chip we can control the production and release of Ag(i) ions in solution and we can thus modulate the activation of the DNA-based switch. Of note, in this case we are unable to control the density of Ag(0) on the chip's surface. Despite this, we can gradually modulate the release of Ag(i) ions by varying the potential step used during the oxidative scan observing a behaviour comparable to that observed with Hg(ii)-activated switches (Fig. S4†).

**Fig. 3:**
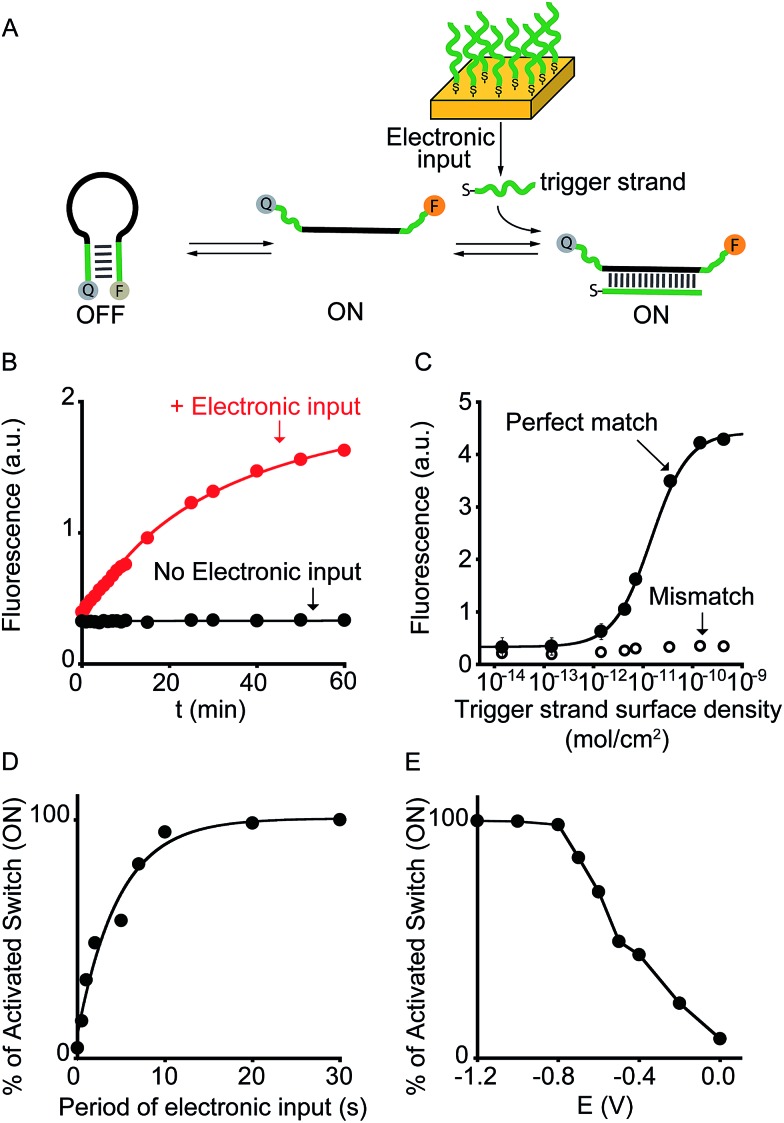
Electronic control of a DNA-responsive nanoswitch. (A) We employed here an optically labeled stem-loop molecular beacon that in the presence of a specific DNA sequence will open to give a fluorescence output.^17^ (B) We can electronically trigger such DNA-based switch by applying a constant reductive potential (–1.2 V *vs.* Ag/AgCl) to a gold chip coated with a thiol-labeled input-strand. The electronic input promotes the reduction of the thiol–gold linkage thus leading to the release of the target strand in solution and to the subsequent switch's opening. (C) We can finely modulate the percentage of activated switches by varying the surface density of the input strand (see also Fig. S5†) and (D) by varying the period of electronic input (from 0 to 30 s) (see also Fig. S6†). (E) Similarly, by changing the intensity of the applied voltage (from –1.2 to 0.0 V *vs.* Ag/AgCl) we can control the amount of input-strand released from the surface and thus the fraction of activated switches (see also Fig. S7†). Here fluorescence measurements were performed in 50 mM sodium phosphate, 150 mM NaCl, pH 7.0 at 45 °C containing the DNA responsive switch (10 nM). We note that 45 °C was chosen considering as the best temperature to achieve a good sensitivity and a high signal-to-noise ratio (see Fig. S8†).

To demonstrate the broad implication of our approach we demonstrate here that we can electronically control the activation of other more general DNA-based nanoswitches. More specifically, we have employed a classic DNA-based nanoswitch (*i.e.* a DNA strand adopting a stem-loop structure) whose conformational change can be induced by a specific DNA strand complementary to the loop sequence (Fig. 3A).^17^ To electronically induce the opening of this stem-loop nanoswitch we have used a thiol-modified DNA strand (input-strand) and we have deposited it on the surface of a gold chip through spontaneous thiol–gold self-assembly reaction.^16^ By applying a constant reductive potential (–1.2 V *vs.* Ag/AgCl) on the gold electrode surface we can electronically induce the reduction of the thiol–gold bond and the release of the input-strand.^18^ This allows to control the activation of the DNA-based nanoswitch (Fig. 3B). Of note, using the same input-coated chip but without applying the electronic input, we observe no activation of the nanoswitch (Fig. 3B, black curve). The electronic activation of this DNA-nanoswitch is also highly tunable and controllable. For example, by varying the concentration of the input-strand used during the coating step we can modulate the input-strand surface density (from 4.3 ± 0.3 × 10^–10^ mol cm^–2^ to 1.4 ± 0.4 × 10^–14^ mol cm^–2^).^19^ This allows to achieve a gradual electronic activation of the nanoswitch (Fig. 3C). We also note that by depositing an analogue input-strand (non-complementary to the loop sequence) and under the same conditions (*i.e.* same surface density and electronic input) we do not observe any activation of the DNA nanoswitch (Fig. 3C, mismatch).

We can also modulate the fraction of activated nanoswitches by varying the time of applied potential on the chip's surface (period of electronic input).^18^ For example, by gradually changing the period of applied potential from 0.5 to 30 s we can modulate the percentage of activated nanoswitches from 15.7 ± 0.2% to 100 ± 3%, respectively (Fig. 3D). Similarly, because the electron transfer rate efficiency that leads to the input-strand release depends on the electronic input, we can modulate the percentage of activated nanoswitches by varying the applied potential. We demonstrate this by using different applied potentials over the same input-strand surface density (4.3 ± 0.3 × 10^–10^ mol cm^–2^) and maintaining a fixed electronic input period (10 s). While applied potentials more negative than –0.8 V *vs.* Ag/AgCl leads to a complete activation of the nanoswitch, less reductive potentials results in a gradual decrease of the fraction of activated nanoswitches from 84 ± 3% (–0.7 V) to 8 ± 2% (0.0 V) (Fig. 3E). Both the examples shown above demonstrate that the electronic approach proposed here could achieve a gradual and quantitative activation of DNA nanodevices.

**Fig. 4:**
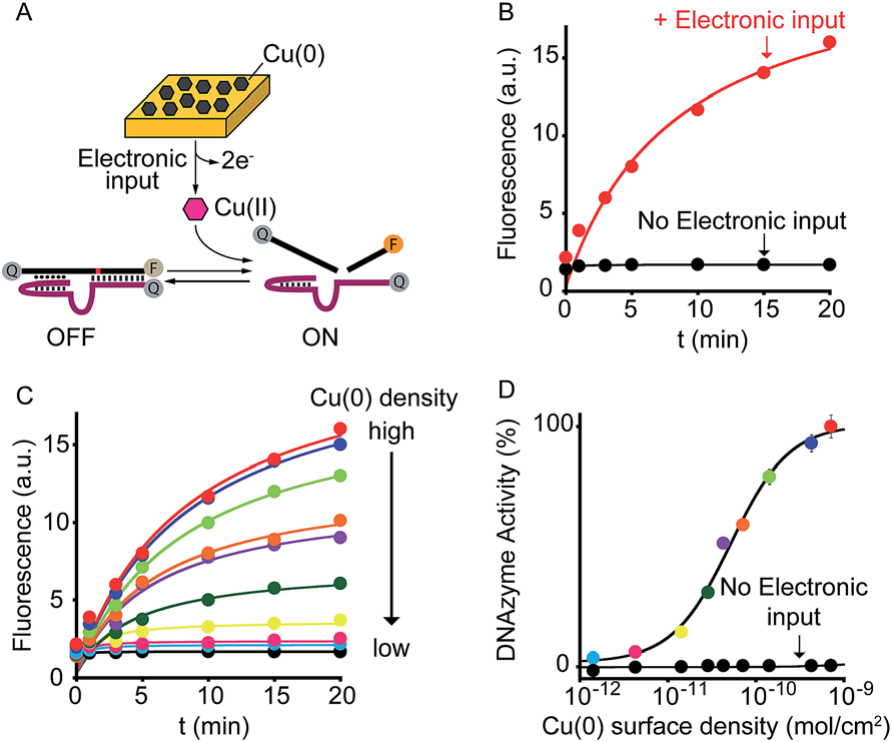
Electronic control of DNAzymes activity. (A) We employed here a DNAzyme with a self-cleavage activity that is activated only in the presence of Cu(ii) ions. (B) By applying a ramp of potential to a gold chip coated with Cu(0) (from –0.65 to –0.4 V *vs*. Ag/AgCl) we can release the molecular trigger (*i.e.* Cu(ii)) and thus control the activation of the DNAzyme. (C) By rationally varying the amount of Cu(0) coated on the gold surface we can finely modulate the activation of the DNAzyme. Of note, control experiments performed under the same experimental conditions but in the absence of the electronic input do not lead to any significant activation of the DNAzyme. Shown are fluorescence time-course experiments performed immediately after applying the electronic input. (D) The end-point values have been used to construct the activation curve shown. Colors correspondence has been used to better identify each curve. Here fluorescence measurements were performed in 1.5 M NaCl, 50 mM HEPES (4-(2-hydroxyethyl)-1-piperazineethanesulfonic acid), pH 7.0 containing the Cu(ii)-responsive DNAzyme.

As a further demonstration of how electrochemistry can improve the current toolkit of possible inputs in the field of DNA-based nanotechnology, we also demonstrate here the possibility to regulate, using an electronic input, the activity of nucleic acid enzymes (*i.e.* DNAzymes). DNAzymes are naturally occurring or *in vitro* selected RNAs or DNAs that catalyze specific chemical reactions. We employed here a well-known DNAzyme (developed by Breaker and coworkers) displaying a Cu(ii)-induced nuclease activity.^20^ The functionality of this DNAzyme can be easily followed by fluorescence measurement by optically labelling the two DNA strands composing the DNAzyme (Fig. 4A and S9†).^21^ More specifically, the two strands are labelled with a fluorophore and a quencher and an increase in fluorescence signal is observed as a result of the Cu(ii)-triggered self-cleaving activity. To electronically control such Cu(ii)-dependent DNAzyme we have used here a gold chip coated with a film of Cu(0). By applying on the surface of this electrode a ramp of potential from –0.65 V to –0.4 V (*vs.* Ag/AgCl) we can electronically promote the release of Cu(ii) ions^22^ in a controlled fashion (Fig. S10†) and thus trigger the DNAzyme activity (Fig. 4B, red curve). Also in this case, under the same experimental conditions but in the absence of the electronic input, we do not observe any significant DNAzyme activation (Fig. 4B, black curve). Moreover, by controlling the density of Cu(0) coated on the gold chip (from 7.1 ± 0.6 × 10^–10^ mol cm^–2^ to 1.4 ± 0.3 × 10^–12^ mol cm^–2^) we can finely modulate the percentage of DNAzyme activation (Fig. 4C and D).

## Conclusions

A limitation in the recently growing field of DNA nanotechnology is associated with the fact that DNA-based nanodevices or nanostructures can be controlled and regulated only by a restricted class of molecular cues (*i.e.* inputs) thus limiting the possibility to achieve an efficient hierarchical control of DNA nanodevices. In response to this, here we have demonstrated the possibility to use electronic inputs to rationally control and regulate DNA-based nanoswitches and nanodevices. We have demonstrated this approach with four model DNA-based nanodevices that are representative of a larger class of conformational-switching and DNA-based enzymes. Of note, each of these model systems is activated by a specific and different input cue that ranges from heavy metal ions (Hg(ii), Ag(i) and Cu(ii)) to a specific oligonucleotide strand.

To electronically activate these DNA-based nanodevices we have used as electronic input a voltage potential applied at the surface of an electrode chip. Such applied potential promotes an electron-transfer reaction at the electrode-solution interphase leading to the release of a molecular input from the electrode surface that ultimately triggers the DNA-based nanodevice in solution. By varying the electronic input we demonstrate that we can rationally modulate the activation of the nanodevices in a highly controllable fashion.

The possibility to use electronic inputs as a way to control DNA-based nanodevices together with the low-cost and possible miniaturization of electrochemical instruments represents an important advancement that allows to expand the available toolbox to be used in the field of DNA nanotechnology thus opening the future to new and exciting avenues. Compared to other examples where DNA-based conformational change is triggered solely through the external addition of an input cue,^23,24^ we believe our approach could be used to introduce additional control over the formation and functionality of DNA nanostructures with an unprecedented hierarchical control.

Our approach could for example prove useful in cases where the intervention of external operator should be avoided. In such cases, a programmable potential application over an electrode chip might be used to release the triggering input in a completely operator-free fashion. We also note that while we have reported here a limited number of electronically-released inputs, we can easily envision the possibility to use such approach to release or activate other molecular inputs through redox reactions.

## Experimental section

### Reagents

Reagent-grade chemicals, including HgCl_2_, AgNO_3_, CuNO_3_, HEPES, sodium phosphate, MOPS, NaCl, HCl (all from Sigma-Aldrich, St Louis, Missouri) were used without further purifications.

HPLC purified oligonucleotides were purchased from Biosearch Technologies (Risskov, Denmark) and IBA GmBH (Göttingen, Germany) and employed without further purification. In this work four different systems were employed. The following oligos modified and non-modified were used for each system:

#### (1) Hg(ii)-responsive nanoswitch (see Fig. 2 and S1†)

The DNA-based switch triggered by mercury(ii) ions has been optimized and characterized elsewhere.^15^ The oligo is internally labeled with FAM (5-carboxyfluorescein) and BHQ-1 (black hole quencher 1) and has the following sequence:

5′-GCATTGTCACTGTCC GTCGAG T(BHQ1)*TTTGTTT GTTGG* T(FAM) CTCGAC *CCTTCTTTCTTA*-3′

#### (2) Ag(i)-responsive nanoswitch (see Fig. S3†)

The DNA-based switch triggered by Ag(i) ions has been optimized and characterized elsewhere.^15^ The oligo is internally labeled with AF680 (Alexa Fluor 680) and BHQ-2 (black hole quencher 2) and has the following sequence:

5′-TTTTATTTAATTATA TTATTAAT T (BHQ2) *CCTACTT TCATC* T (AF680) ATTAATAA *CATCAAACTACC*-3′

#### (3) DNA-responsive nanoswitch (see Fig. 3 and S8†)

The DNA-responsive nanoswitch is a molecular beacon containing a 5-base stem and it is labeled with FAM (5-carboxyfluorescein) and a BHQ-1 (black hole quencher 1) and has the following sequence:

5′-(FAM) A CTCAC *TGTGCTGACCAGTCTCT* GTGAG G(BHQ1)-3′

In the sequence above the underlined bases represent the stem portion, while the italic bases represent the recognition element of the binding-state.

#### (4) Cu(ii)-responsive DNAzyme (see Fig. 4 and S9†)

The Cu(ii)-responsive DNAzyme has been also characterized elsewhere.^21^ The system is composed of two different strands. The first strand is labeled with AF680 (Alexa Fluor 680) and BHQ-2 (black hole quencher 2) and the second strand is labeled with BHQ-2 (black hole quencher 2). The sequences of the two strands are given below:

Strand 1: 5′-(BHQ2) AGC TTC TTT CTA ATA CGG CTT ACC (AF680)-3′

Strand 2: 5′-(BHQ2) GGT AAG CCT GGG CCT CTT TCT TTT TAA GAA AGA AC-3′

See the cartoon in Fig. 4 to clarify the copper-binding site and mechanism for obtaining information on the activity of the DNAzyme.

### Electronic activation of DNA-based nanodevices

In this work we report the use of electronic inputs to activate all the four different DNA-based nanodevices described above. All experiments were performed using a portable PalmSens potentiostat instrument connected to a laptop. Briefly, the molecular input of each DNA-based nanodevice has been deposited onto the surface of a screen printed disposable electrode. Through an electrochemical input (applied potential) we have released the molecular input in a controlled way and we have thus triggered the DNA-based nanodevice. The occurred activation of the DNA-based nanodevice has been followed through fluorescent measurement. The detailed experimental procedure employed for each system is described in the ESI document.†

### Standard binding curves

Standard binding curves were obtained for each system employed in this work by adding at increasing concentrations the specific molecular input and measuring the fluorescence signal. For each system the same buffer solution used for the electronic activation experiment (see ESI†) and the same fluorometer parameters were used. The targets used for each system are the following: HgCl_2_ (as a source of Hg(ii) ions) for Hg(ii)-responsive nanoswitch, AgNO_3_ (as a source of Ag(i) ions) for Ag-responsive nanoswitch, the thiol-labeled strand target for DNA-responsive nanoswitch, CuNO_3_ (as a source of Cu(ii) ions) for the Cu(ii)-responsive DNAzyme.

The observed fluorescence, *F*_[target]_, was fitted using the following four parameter logistic equation^25^ *F*_[target]_ = *F*_min_ + (*F*_max_ – *F*_min_)[[target]^*n*_H_^/([target]^*n*_H_^ + *K*_1/2_^*n*_H_^)]where, *F*_min_ and *F*_max_ are the minimum and maximum fluorescence values, *K*_1/2_ is the equilibrium target concentration at half-maximum signal, *n*_H_ is the Hill coefficient, and [target] is the concentration of the target added. This model is not necessarily physically relevant, but it does a good (empirical) job of fitting effectively binding curves such as those we obtain for most of our systems employed in this work.
