# Supplementary material for: Electronic control of DNA-based nanoswitches and nanodevices
Source: Chem Sci. 2015 Nov 12;7(1):66–71. doi: 10.1039/c5sc03694a (PMC5508672; doi:10.1039/c5sc03694a)
Supplement: Supplementary file 1 [file SC-007-C5SC03694A-s001.pdf]

Electronic Supplementary Material (ESI) for Chemical Science.
This journal is © The Royal Society of Chemistry 2015

Electronic control of DNA-based nanoswitches and nanodevices

*Simona Ranallo[1], Alessia Amodio[1,2], Andrea Idili[1], Alessandro Porchetta[1], Francesco Ricci[1,]**

[1] Chemistry Department, University of Rome, Tor Vergata, Rome, Italy.

[2] PhD School of Nanotechnology, Department of Physics, University of Trieste, Trieste, Italy

1-SUPPORTING METHODS

Electronic activation of DNA-based nanodevices:

In this work we report the use of electronic inputs to activate the four different systems described above. All experiments were performed using a portable PalmSens potentiostat instrument connected to a laptop. The following experimental procedure were used for each system:

Electronic activation of Hg(II)-responsive nanoswitch (see Fig. 2, S1 and S2):

For the electrochemical activation of Hg(II)-responsive nanoswitch (see Fig. 2 and S1) the following procedure was employed. First, we used gold-based screen printed electrode chips produced in-house as reported elsewhere.[2] Briefly, the electrodes were printed with a 245 DEK (Weymouth,UK) screen printing machine, and using the following conductive inks: graphite-based, gold-based and silver-based. An insulating ink was also used to produce the electrodes. The inks were printed on a polyester flexible film (Autostat HT5). Each gold-based screen printed electrode chip contains three printed separated portions that act as the working (gold-based ink), the reference (silver-based ink) and the counter electrode (the carbon-based ink). The diameter of the working electrode was 0.3 cm, which resulted in an apparent geometric area of 0.07 cm^2.

We have coated the gold working electrode with a film of Hg(0) by immersing the electrode in a 5 mL solution 0.05 M HCl containing $HgCl_2$ (at different concentrations) under stirring condition. The coating was performed by applying a fixed potential of 0.2 V vs Ag/AgCl for 240 seconds. To modulate the density of Hg(0) coated on the surface of the working electrode we have employed

different concentrations of $HgCl_2$ during the deposition step (from 0.01 μM to 10 mM, see Fig. 2 and Fig. S2). The density of Hg(0) (Γ) on the electrode surface was determined from the integrated charge under the oxidation peak in SWV scans using the following equation:

$$\Gamma = Q/nFA$$

Where Q is the integrated charge under the reduction peak, n is the number of electrons transferred (n = 2 for Hg(II)), F is the Faraday's constant, and A is the real electrode area.

The screen-printed electrode chip was then thoroughly rinsed with distilled water.

To perform the electronic activation of the mercury-responsive nanoswitch we then placed a 100 μL drop of a solution of 50 mM sodium phosphate, 150 mM NaCl, pH 7.0 containing the mercury-responsive nanoswitch (10 nM) onto the screen printed electrode chip placed in a horizontal position. The electronic activation was achieved by applying a ramp of potential from 0.2 to 0.65 V using a Square Wave Voltammetry (SWV) technique with the following operative parameters: E_{step} = 0.004V, Amplitude = 0.04 V, Frequency = 15Hz. At the end of the SWV procedure, the 100 μL solution containing the nanoswitch was transferred into a micro-cuvette for fluorescence measurement using an excitation wavelength of 480 (± 5) nm and acquisition at 517 (± 5) nm at 25°C.

For control experiments (no electronic input, see Fig. 2), we have used the same exact procedure described above except that during the electrochemical activation the electrode was not connected to the potentiostat.

Electronic activation of Ag(I)-responsive nanoswitch (see S4):

For the electronic activation of Ag(I)-responsive nanoswitch (see Fig. S3) the following procedure was employed. First, we used silver-based screen printed electrode chips produced in-house. These silver screen-printed electrode chips are produced as reported above for gold screen-printed electrode chips with the only difference that a silver-based ink was used to print the working electrode. Because the working electrode of these screen-printed electrode chips already contain silver(0) particles, the deposition step employed above for mercury-responsive nanoswitches is not needed. Before use the screen-printed electrode chips were throughly rinsed with distilled water. To perform electronic activation of the silver-responsive nanoswitch we placed a 100 μL drop of a solution of 10 mM Mops (3-(N-morpholino)propanesulfonic acid), pH 7.0 containing the silver-

responsive nanoswitch (50 nM). The electronic activation was achieved by applying a ramp of potential from -0.2 to +0.6 V using SWV with the following operative parameters: E_{step} = 0.004V, Amplitude = 0.04V, Frequency = 15Hz. At the end of the SWV procedure, the 100 μL solution containing the nanoswitch was then transferred into a micro-cuvette for fluorescence measurement using an excitation wavelength of 680 (± 5) nm and acquisition at 702 (± 5) nm at 25°C.

Electronic activation of DNA-responsive nanoswitch (see Fig. 3, S6, S7):

For the electrochemical activation of DNA-responsive nanoswitch (see Fig. 3 and S5) the following procedure was employed. First, we used gold screen-printed electrode chips produced in-house (see above). We have deposited a thiol-labeled sequence complementary to the loop of the DNA-responsive nanoswitch (sequence: 5'-C6-thiol-CAGAGACTGGTCAGCACA-3') onto the gold working-electrode surface. To do so, a 100 μL solution of 1 M NaCl, 10 mM sodium phosphate, pH 7.0, containing the complementary sequence was placed onto the gold working-electrode and the spontaneous formation of a self-assembled monolayer due to gold-thiol reaction was allowed for 24 hours. To modulate the amount of the complementary sequence deposited onto the surface of the working electrode we have employed different concentrations during the deposition step (from 0.5 μM to 10 μM). The electrodes were then thoroughly rinsed with distilled water.

To perform electronic activation of the DNA-responsive nanoswitch we placed a 100 μL drop of a solution of 50 mM sodium phosphate, 150 mM NaCl, pH 7.0 containing the DNA-responsive nanoswitch (10 nM). The electronic activation was achieved by applying a fixed potential of -1.2 V for 30 seconds. At the end of this electrochemical procedure, the 100 μL solution containing the nanoswitch was transferred into a micro-cuvette for fluorescence measurement using an excitation wavelength of 480 (± 5) nm and acquisition at 517 (± 5) nm (Fig. 3B) at 45°C.

To modulate the amount of complementary sequence electronically released from the electrode surface we have employed different applied potentials (see Fig. 3E and S7) and we have also varied the period of the applied potential (-1.2 V) from 0 to 30 seconds (see Fig. 3D and S6).

For control experiments (no electronic input, see Fig. 3B), we have used the same exact procedure described above except that during the electronic activation the electrode was not connected to the potentiostat.

For control experiments where we electronically released from the electrode surface a non-complementary sequence (instead of a complementary sequence, see "mismatch" curve, Fig. 3C),

we have used the same exact procedure described above except that during the deposition step a thiol-labeled non-complementary sequence (sequence: 5'-C6-thiol-TTCGTATTATATTAATAA-3') was used.

Electronic activation of Cu(II)-responsive DNAzyme (see Fig. 4 and S10):

For the electronic activation of Cu(II)-responsive DNAzyme (see Fig. 4 and S10) the following procedure was employed. First, we used carbon screen-printed electrode chips produced in-house (see above). These carbon screen-printed electrode chip are produced as reported above for gold screen-printed electrode chips with the only difference that a carbon-based ink was used to print the working electrode. We have deposited Cu(0) on the working electrode of the carbon screen printed electrode chip by dipping the electrode in 5 mL of a solution 0.05 M HCl containing $CuNO_3$ (at different concentrations) under stirring conditions. The deposition was performed by applying a fixed potential of -1.0 V vs Ag/AgCl for 90 seconds. To modulate the amount of Cu(0) deposited onto the surface of the working electrode we have employed different concentrations of $CuNO_3$ during the deposition step (from 1 to 100 µM, see Fig. S10).

The density of Cu(0) (Γ) on the electrode surface was determined from the integrated charge under the oxidation peak in SWV scans using the following equation:

$$\Gamma = Q/nFA$$

Where Q is the integrated charge under the reduction peak, n is the number of electrons transferred (n = 2 for Cu(II)), F is the Faraday's constant, and A is the real electrode area.

The screen-printed electrode chip was then rinsed with distilled water. To perform electronic activation of the Cu(II)-responsive DNAzyme we then placed a 100 µL drop of a solution of 1.5 M NaCl, 50 mM HEPES (4-(2-hydroxyethyl)-1-piperazineethanesulfonic acid), pH 7.0 containing the Cu(II)-responsive DNAzyme at a concentration of 10 nM Strand 1 and 20 nM Strand 2 (previously prepared as reported elsewhere).[1] The electrochemical activation was achieved by applying a ramp of potential from -0.65 to -0.4 V using SWV with the following operative parameters: E_{step} = 0.001 V, Amplitude = 0.01 V, Frequency = 10 Hz. At the end of the SWV procedure, the 100 µL solution containing the DNAzyme was then transferred into a micro-cuvette containing 50 µM ascorbate for fluorescence measurement using an excitation wavelength of 680 (± 5) nm and acquisition at 702 (± 5) nm at 23 °C. For control experiments (no electronic input, see Fig. 4B), we have used the same

exact procedure described above except that during the electrochemical activation the electrode was not connected to the potentiostat.

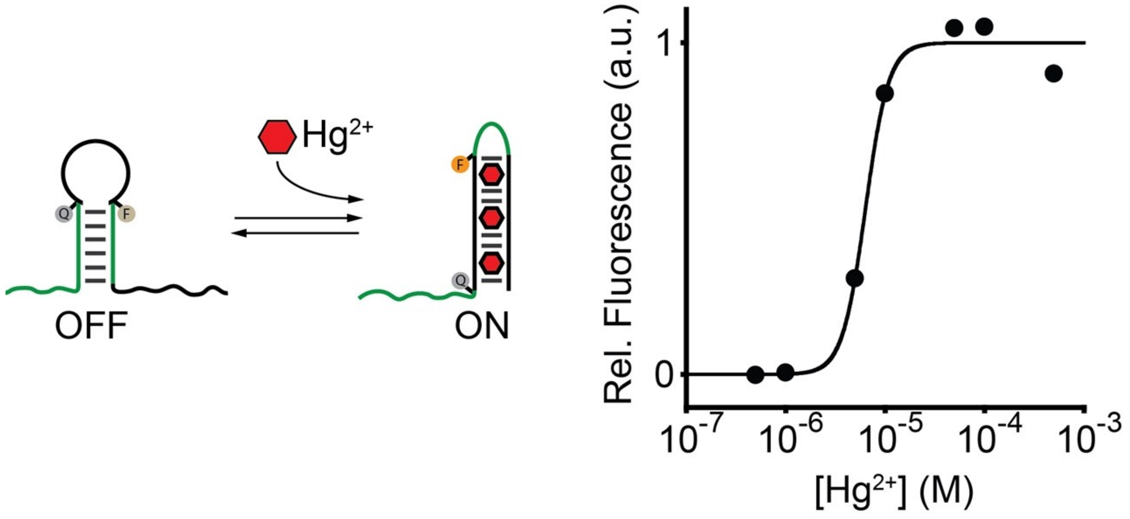

Fig. S1. Binding curves of the Hg(II)-responsive nanoswitch obtained at increasing concentration of $HgCl_2$ in a 100 μL solution of 50 mM sodium phosphate, 150 mM NaCl, pH 7.0 containing the Hg(II)-responsive nanoswitch (10 nM). Binding curve is shown as normalized signals.

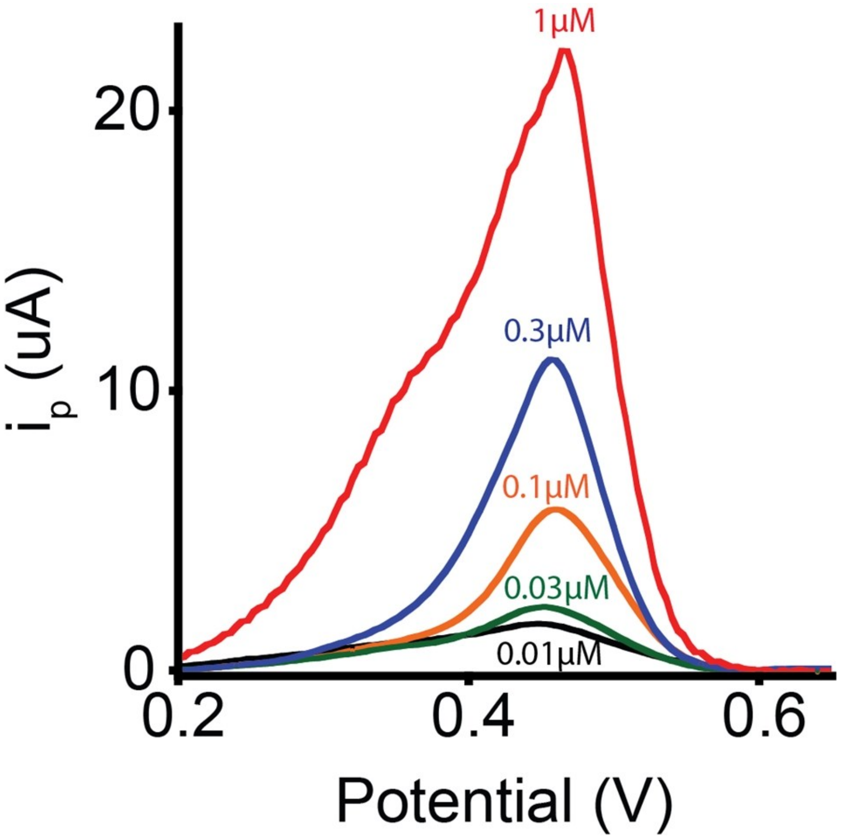

Fig. S2. To modulate the density of Hg(0) coated onto the surface of the working electrode of the gold screen printed electrode chip we have employed different concentrations of $HgCl_2$ during the deposition step (from 0.01 μM to 10 mM) by dipping the electrode in 5 mL of a solution 0.05 M HCl containing $HgCl_2$ under stirring conditions and by applying a fixed potential of 0.2 V vs Ag/AgCl for 240 seconds. The oxidation and the release of the mercury ions (Hg(II)) was achieved by applying a ramp of potential from 0.2 to 0.65 V using SWV with the following operative parameters: E_{step} = 0.004V, Amplitude = 0.04V, Frequency = 15Hz, in 100 μl of a solution 0.05 M HCl. Here representative scans at different Hg(0) densities are shown. The current peak is thus directly proportional to the amount of Hg(0) coated on the surface of the electrode thus ultimately resulting in the modulation of the amount of Hg(II) released from the surface.

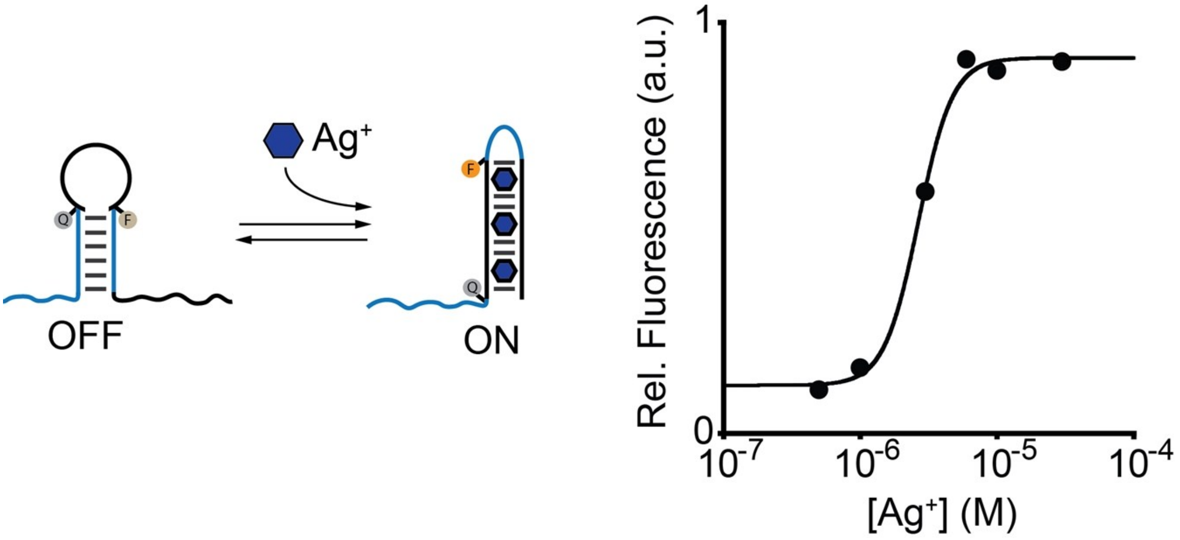

Fig. S3. Binding curves of the Ag(I)-responsive nanoswitch obtained at increasing concentration of $AgNO_3$ in a 100 μL solution of 10 mM Mops (3-(N-morpholino)propanesulfonic acid), pH 7.0 containing the Ag(I)-responsive nanoswitch at a concentraton of 50 nM. Binding curves are shown as normalized signals.

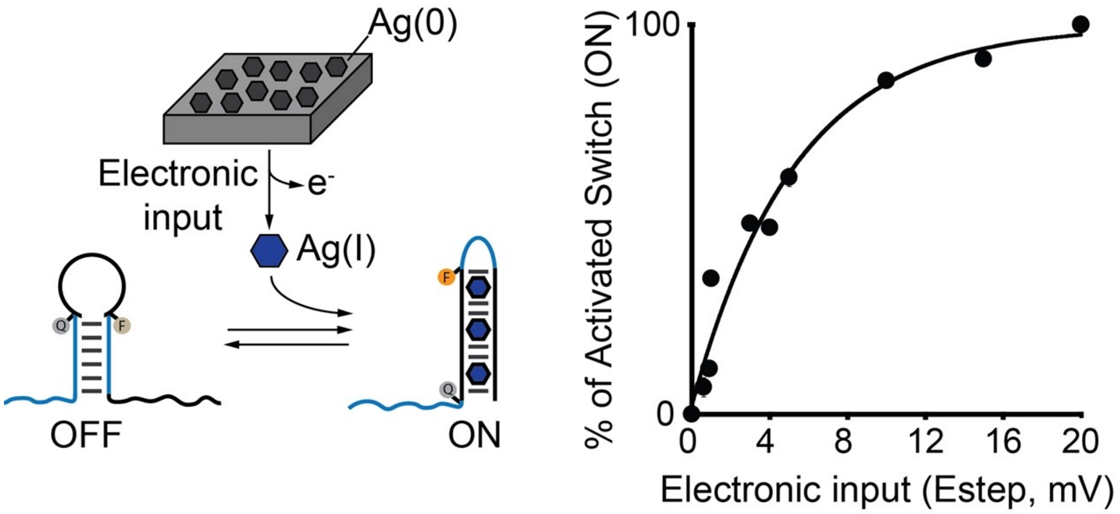

Fig. S4. Electronic activation of Ag(I)-responsive nanoswitch. (left) Schematic of the Ag(I)-responsive nanoswitch system. (right) The electronic activation was achieved by applying a ramp of potential from -0.2 to +0.6 V using SWV (see above for details). We performed an electronic modulation of the Ag(I)-responsive nanoswitch through the variation of a parameter (E_{step}) of the SWV technique obtaining a similar behavior to that observed with the Hg(II)-responsive (see Fig. 2). Here fluorescence measurements were performed in 10 mM Mops (3-(N-morpholino)propanesulfonic acid), pH 7.0 at 25 °C with the Ag(I)-responsive nanoswitch at a concentration of 50 nM.

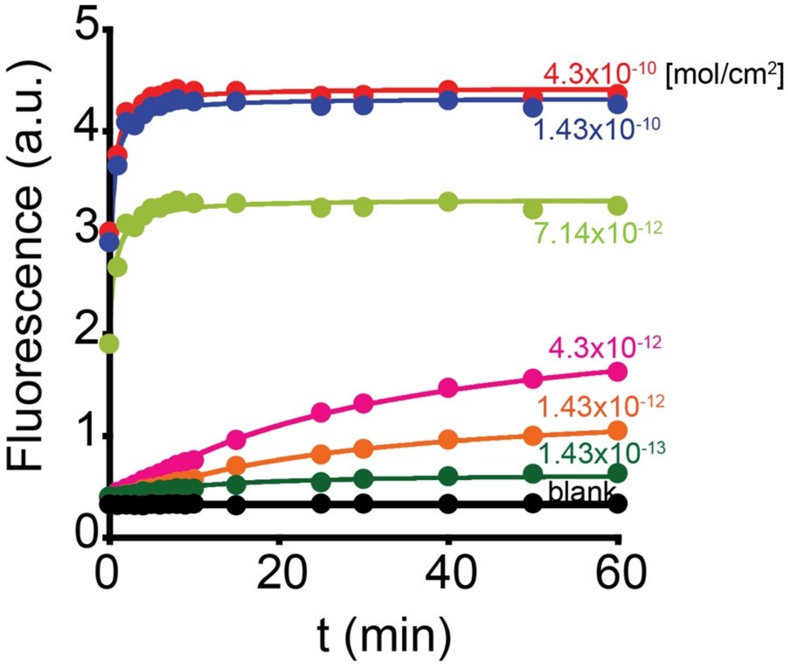

Fig. S5. We can modulate the activation of a DNA-responsive switch by varying the density of the input strand coated on the gold-electrode chip. Here we applied a constant potential (-1.2 V vs Ag/AgCl) to a gold chip coated with different densities of the target strand (see Fig. 3) for a period of 30 seconds to a 100 µl drop of a solution of 50 mM sodium phosphate, 150 mM NaCl, pH 7.0 containing the DNA-responsive nanoswitch (10 nM). The 100 µL solution containing the nanoswitch was then transferred into a micro-cuvette for fluorescence measurement using an excitation wavelength of 480 (± 5) nm and acquisition at 517 (± 5) nm at 45°C. Fluorescence was measured intermittently over a period of 60 minutes. In Fig. 3C the final fluorescence signals obtained in this experiment have been plotted towards the period of electronic input used.

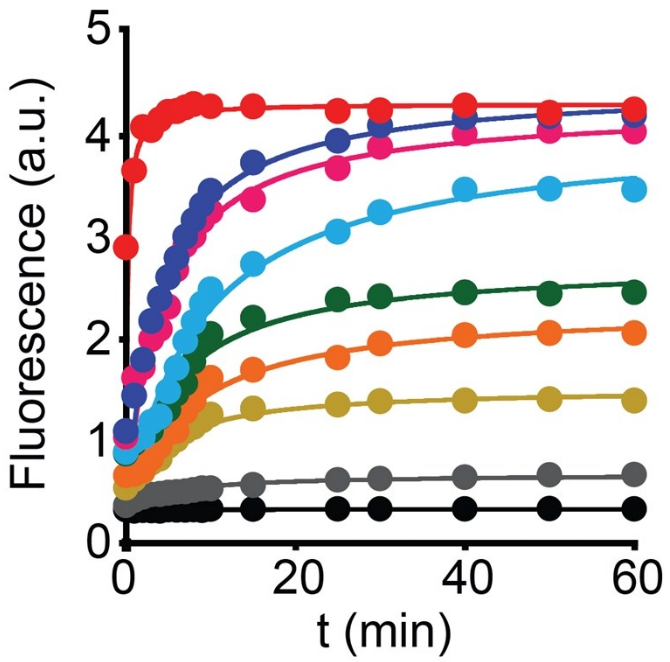

Fig. S6. We can modulate the activation of a DNA-responsive switch by varying the period of application of the electronic input (time of applied potential on the chip's surface). Here we applied a constant potential (-1.2 V vs Ag/AgCl) to a gold chip coated with the target strand (see Fig. 3) for a period ranging from 30 seconds (red curve) to 0 seconds (blak curve) to a 100 µl drop of a solution of 50 mM sodium phosphate, 150 mM NaCl, pH 7.0 containing the DNA-responsive nanoswitch at a concentration of 10 nM. The 100 µL solution containing the nanoswitch was then transferred into a micro-cuvette for fluorescence measurement using an excitation wavelength of 480 (± 5) nm and acquisition at 517 (± 5) nm at 45°C. Fluorescence was measured intermittently over a period of 60 minutes. In Fig. 3D the final fluorescence signals obtained in this experiment have been plotted towards the period of electronic input used.

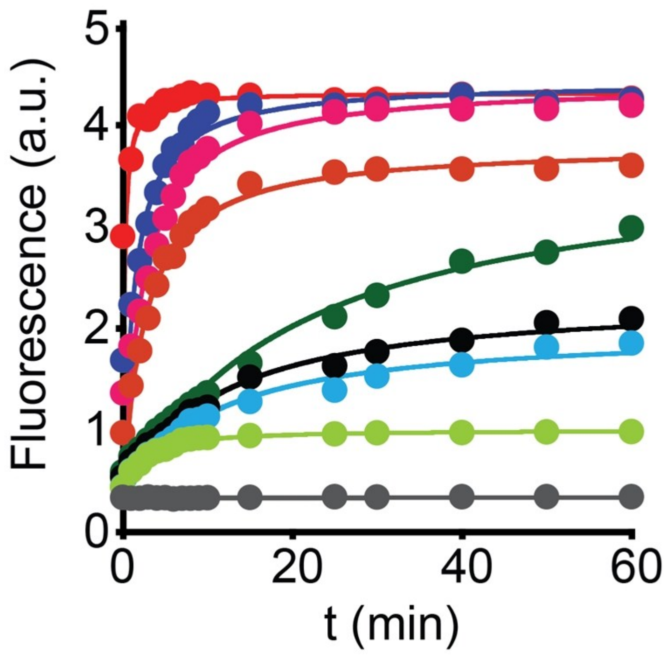

Fig. S7. We can modulate the activation of a DNA-responsive switch by varying the electronic input (applied potential on the chip's surface). Here we applied varying potentials (from -1.2 to 0.0 V vs Ag/AgCl) to a gold chip coated with the target strand at a constant density of 4 x 10^{-10} mol/cm^2 (see Fig. 3) for a period of 30 seconds to a 100 µl drop of a solution of 50 mM sodium phosphate, 150 mM NaCl, pH 7.0 containing the DNA-responsive nanoswitch at a concentration of 10 nM. The 100 µL solution containing the nanoswitch was then transferred into a micro-cuvette for fluorescence measurement using an excitation wavelength of 480 (± 5) nm and acquisition at 517 (± 5) nm at 45°C. Fluorescence was measured intermittently over a period of 60 minutes. In Fig. 3E the final fluorescence signals obtained in this experiment have been plotted towards the applied potential.

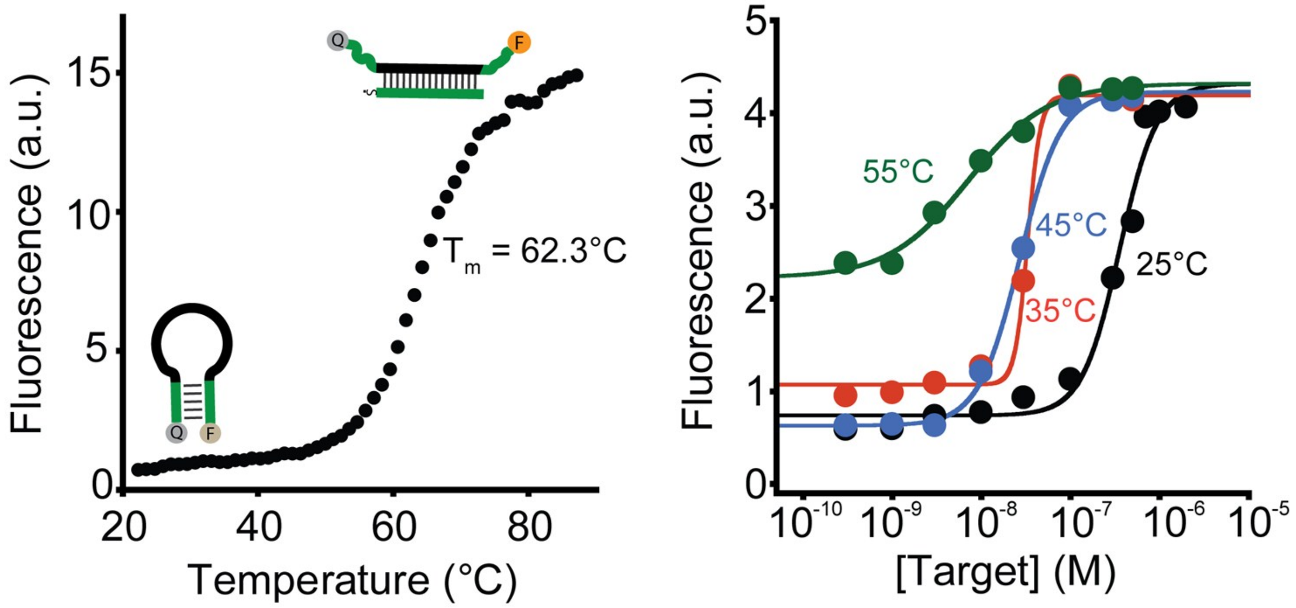

Fig. S8. The DNA-responsive nanoswitch employed in this work has a melting temperature of 62.3 °C (left). We have characterized the response of this DNA-responsive nanoswitch to increasing concentrations of a target DNA complementary at different temperatures to evaluate the optimal conditions for testing (right). We found out that at 45°C we achieve the best sensitivity and the highest signal-to-noise ratio. Here the melting curve has been obtained by increasing the temperature of the solution by 1°C/min and using a concentration of DNA-responsive nanoswitch of 10 nM. The binding curves were obtained by adding increasing concentrations of the thiol-modified complementary input strand in a 100 μL solution of 50 mM sodium phosphate, 150 mM NaCl, pH 7.0 at 45°C containing the DNA-responsive nanoswitch at a concentraton of 10 nM.

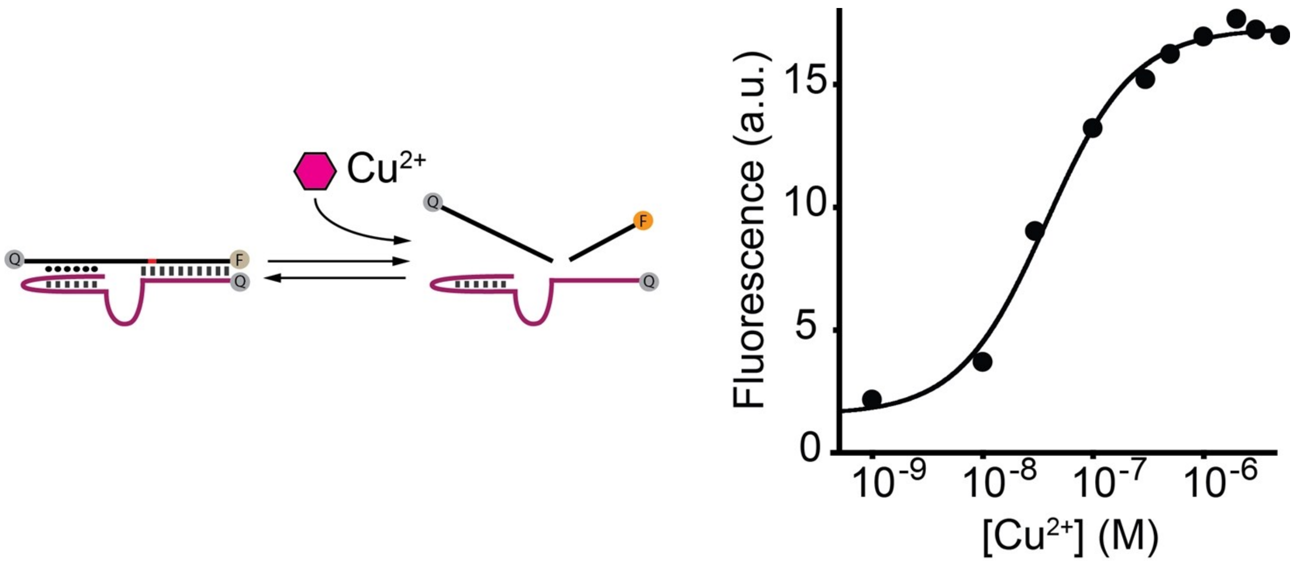

Fig. S9. Binding curves of the Cu(II)-responsive DNAzyme obtained at increasing concentration of $CuNO_3$ in a 100 µL solution of 1.5 M NaCl, 50 mM HEPES (4-(2-hydroxyethyl)-1-piperazineethanesulfonic acid), pH 7.0 containing the Cu(II)-responsive DNAzyme at a concentration of 10 nM Strand 1 and 20 nM Strand 2 (prepared as described above), pH 7.0 at 23 °C.

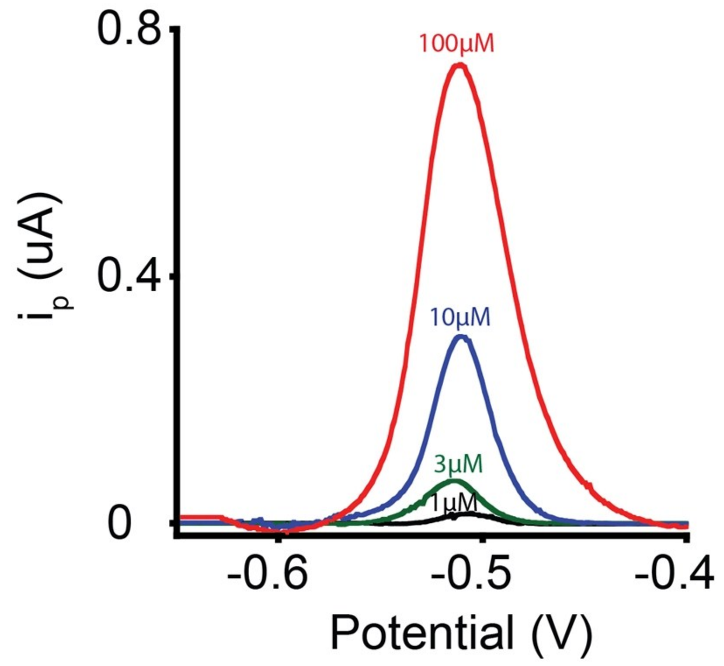

Fig. S10. To modulate the amount of Cu(0) deposited onto the surface of the working electrode of the carbon screen printed electrode chip we have employed different concentrations of $CuNO_3$ during the deposition step (from 1µM to 100 µM), by dipping the electrode in 5 mL of a solution 0.05 M HCl containing $CuNO_3$ in stirring condition and by applying a fixed potential of -1.0 V vs Ag/AgCl for 90 seconds. The oxidation and the release of the mercury ions (Cu(II)) was achieved by applying a ramp of potential from -0.64 to -0.4 V (shown is only a selected region of the scan) using Square Wave Voltammetry with the following operative parameters: E_{step} = 0.001 V, Amplitude = 0.01V, Frequency = 10 Hz, in 100 µl of a solution 0.05 M HCl.

REFERENCES:

1. (a) L. Zhang, Y. Zhang, M. Wei, Y. Yi, H. Li, S. Yao, *New J. Chem.* 2013, **37**, 1252-1257. (b) S. Shimron, J. Elbaz, A. Henning, I. Willner, *Chem. Commun.* 2010, **46**, 3250. (c) J. Liu, Y. Lu, *J. Am. Chem. Soc.* 2007, **129**, 9838-9839.

2. B. Esteban Fernández de Ávila, H.M. Watkins, J.M. Pingarrón, K.W. Plaxco, G. Palleschi, F. Ricci, *Anal. Chem.* 2013**, 85**, 6593–6597.
